# Spatial variation and risk factors of malaria and anaemia among children aged 0 to 59 months: a cross-sectional study of 2010 and 2015 datasets

**DOI:** 10.1038/s41598-022-15561-4

**Published:** 2022-07-07

**Authors:** Jecinta U. Ibeji, Henry Mwambi, Abdul-Karim Iddrisu

**Affiliations:** 1 School of Mathematics, Statistics and Computer Science, University of KwaZulu Natal, Durban, South Africa; 2 School of Science, Mathematics and Statistics, University of Energy and Natural Resources, Sunyani, Ghana

**Keywords:** Medical research, Diseases

## Abstract

Malaria and anaemia are common diseases that affect children, particularly in Africa. Studies on the risk associated with these diseases and their synergy are scanty. This work aims to study the spatial pattern of malaria and anaemia in Nigeria and adjust for their risk factors using separate models for malaria and anaemia. This study used Bayesian spatial models within the Integrated Nested Laplace Approach (INLA) to establish the relationship between malaria and anaemia. We also adjust for risk factors of malaria and anaemia and map the estimated relative risks of these diseases to identify regions with a relatively high risk of the diseases under consideration. We used data obtained from the Nigeria malaria indicator survey (NMIS) of 2010 and 2015. The spatial variability distribution of both diseases was investigated using the convolution model, Conditional Auto-Regressive (CAR) model, generalized linear mixed model (GLMM) and generalized linear model (GLM) for each year. The convolution and generalized linear mixed models (GLMM) showed the least Deviance Information Criteria (DIC) in 2010 for malaria and anaemia, respectively. The Conditional Auto-Regressive (CAR) and convolution models had the least DIC in 2015 for malaria and anaemia, respectively. This study revealed that children in rural areas had strong and significant odds of malaria and anaemia infection [2010; malaria: AOR = 1.348, 95% CI = (1.117, 1.627), anaemia: AOR = 1.455, 95% CI = (1.201, 1.7623). 2015; malaria: AOR = 1.889, 95% CI = (1.568, 2.277), anaemia: AOR = 1.440, 95% CI = (1.205, 1.719)]. Controlling the prevalence of malaria and anaemia in Nigeria requires the identification of a child’s location and proper confrontation of some socio-economic factors which may lead to the reduction of childhood malaria and anaemia infection.

## Introduction

Malaria is an acute febrile illness caused by *Plasmodium parasites*, which are transmitted to humans via the bites of infected female *Anopheles mosquitoes*^1^. Nearly half of the world’s population was at risk of malaria in 2020, where children under the age 5 years were mostly affected in sub-Sahara Africa both in terms of mortality and morbidity^1–4^. This has remained a major public health problem notwithstanding it could be treated, prevented and cured^5^. Based on the latest projections from United Nations data, Nigeria, as one of the countries in Western Africa, has a population of approximately 208 million^6^. By this projection, Nigeria has termed the largest community in Africa. Africa bears over 80% of global malaria burden, and Nigeria has about 29% of this burden. Furthermore, Nigeria together with the Democratic Republic of Congo contributes approximately 40% of global burden^7^. In Nigeria, malaria is accountable for up to 60% of outpatients’ visits and 30% of admissions. Also, malaria is believed to contributes to about 11% of maternal death, 25% of infant death, and 30% to under 5 year age mortality^5^. Therefore, the country contribute a big proportion of deaths of mostly children and pregnant women every year in the region^8^. According to a recent report from WHO, Nigeria has 1.3 million cases of malaria out of 3.5 million cases recorded by the highest-burden countries^9^. Though WHO suggested that for global burden of disease to improve, there is an urgent need for a speedy reduction in the incidence of the disease in high-burden countries which unfortunately Nigeria is lagging^9^.

Also, anaemia is another global public health problem that mostly affects children under 5 years of age and pregnant women^10^. It is a condition in which the hemoglobin (Hb) in the blood is lesser than needed by the body for its optimal physiological function^11,12^. Anaemia is one of the complications seen in malaria infection, which contributes to its morbidity and mortality. According to World Health Organization (WHO) report, between 2015 and 2018, anaemia prevalence in children under the age of 5 years with a positive rapid diagnostic test (RDT) was twice that of children with a negative RDT in 21 African countries with moderate to high malaria prevalence^13^. Children who tested positive for malaria had 9% severe anaemia and 54% moderate anaemia. On the other hand, children who tested negative for malaria had only 1% severe anaemia and 31% moderate anaemia^13^. Nigeria has been confirmed to be among the countries in Africa with the highest anaemia prevalence of under aged 5 years children^14,15^. The risk factors of anaemia in children are multifactorial and interrelated in a complex way^16^. In high-income countries, iron deficiency is known to be the major reason for anaemia unlike in low and middle-income countries (LMIC) where another factor like malaria is a major contributor to childhood anaemia especially in Sub-Sahara Africa (SSA)^11,17^.

The success of any healthcare program intervention is determined by a comprehensive and adequate understanding of the multifaceted determinants of the occurrence of diseases and death^8^. Until recently, data information on malaria, anaemia and other childhood diseases in Nigeria has been from clinics and hospitals. But then, these information are just a small portion of the total cases because it has been proven that a large percent of caregivers are conversant with the symptoms of fever associated with malaria, as such, about 80% of fevers are taken care of at home^8,18–20^. Thus, data from the hospital may not be sufficient for estimating the prevalence and determinant factors of malaria and anaemia for appropriate program development^21^. As a replacement, the cross-sectional data from the Malaria Indicator Survey (MIS) was collected to gather information on malaria and anaemia in children under age 5 years.

Nigerian government via the National Control Program, in line with many other non-governmental bodies such as Roll Back Malaria (RBM) through the implementation of (2009–2013) malaria control strategic plan has continued to make obvious efforts in reducing the spread of malaria and associated child death. Also, malaria awareness programs through mass distribution of long-lasting insecticide-impregnated nets (LLINs) within the selected states of the country has been put in place. From 2010 to 2015, there was an obvious reduction in malaria prevalence from 52 to 45% through their effort^5^. Despite these intervention programs, there has been a continuous record of morbidity and mortality from malaria, anaemia and other infectious diseases for children under age 5 years in most developing countries^2,22^.

Therefore, preventing fatal outcome in malaria cases requires recognition of infection, accurate laboratory diagnosis, and prompt therapy^23,24^. Geoadditive latent variable models for binary/ordinal indicators was adopted to analyze the influence of variables of different types on the morbidity among young children in Nigeria^8^. Nevertheless, epidemiology studies focus on analyzing the geographic variation for the severity of the disease. A Multilevel logit model accounting for sampling design were adopted to assess individual, household and community factors associated with malaria infection^25^. On the other hand, Generalized Additive Model (GAM) has been applied to identify the important risk factors that influence the prevalence of childhood malaria in Nigeria^26^. A multivariable hierarchical Bayesian geoadditive model with the inclusion of a spatial effect was used by Robert et al.^11^ to investigate the spatial variation and risk factors of childhood anaemia in four sub-Sahara African countries. While Ugwu and Zewotir^27^ adopted a binary structured additive regression (BSAR) regression to examine the spatial heterogeneity and determinants of childhood anaemia in Nigeria. Studies on the risk factors and relationship between these diseases is scanty, also, no study has compared the differences in their spread for two separate years. Therefore, this study is aimed at investigating the spatial pattern of malaria as well as anaemia and adjusting for risk factors associated with each disease using a separate multivariate hierarchical Bayesian logistic model for each disease.

## Data and method

### Studied data

This study used the 2010 and 2015 data collected in the Malaria Indicator Survey (MIS) carried out in Nigeria. In both years, the sampling frame was obtained from the 2006 Population and Housing Census of the Federal Republic of Nigeria that was conducted by the National Population commission^5,28^. Nigeria as a nation is divided into 37 states administratively including the capital territory and each state is divided into local government areas (LGAs) then each LGA is further divided into localities. For convenience, each locality was subdivided into census enumeration areas (EAs). These EAs from the 2006 EA census frame were used to define the cluster (i.e. primary sampling unit (PSU))^5^.

A two-stage probability sampling was assumed in 2010 and 2015. While in 2010, the two-stage cluster design has a total number of 240 clusters, 83 in the urban areas and 157 clusters in the rural areas. In the end, 239 clusters were used due to intercommunal uproar in one of the clusters. Approximately, a representative sample of 6000 households was selected for the survey, with a minimum target of 920 completed individual women’s interviews per zone. This is for the first stage. In the second stage, by equal probability systematic sampling, an average of 26 households were selected in each cluster. All women from age 15–49 were interviewed, also, children from 6 to 59 months were tested for malaria and anaemia^28^. On the other, in addition to Federal Capital Territory (FCT), 9 clusters (EAs) were selected from each state. Each state was represented in the sample with a total of 333 clusters around the country, 138 in urban areas and 198 in rural areas. From each cluster, 25 households were selected in the second stage by equal probability systematic sampling, while all women age 15–49 were interviewed, and all children age 6–59 months were tested for malaria and anaemia^5^.

Furthermore, a comprehensive inventory of households was carried out in both years. In 2010, the mapping exercise was done from August to September while in 2015 it was carried out from June to July^5,29^. In addition, global positioning system (GPS) receivers were used by NPC listing enumerators to record the coordinates of the 2010 and 2015 NMIS sample clusters respectively.

### Dependent variables

The dependent variables used in this study are the malaria status (presence or absence) and anaemia status (presences of absences) variables. Malaria is a disease spread to a human through the bite of infected anopheles’ mosquito. The presence of malaria antigens discharged from the parasitized red blood cells is detected by the malaria diagnostic test which is a form of immunochromatography test. Both rapid diagnostic and microscopy testing have been approved by World Health Organization (WHO) as procedures for malaria diagnosis. Even though microscopy is recognized as the standard approach for malaria diagnosis, the application is demanding. While microscopy requires an experienced microscopist, a good environment, time etc., RDTs do not need skilled personnel, specialized equipment and long process^2^.

Anaemia is a condition resulting from the decrease or dysfunctional red blood cells in the body. Iron deficiency is known as a common cause of anaemia but in developing countries, malaria as one of the infectious diseases is attributed to anaemia disease^5^. As recommended by WHO, children from age 6 to 59 months are said to be anaemic if the Hb concentration level is below 11.0 g/dl: those within age 5 to 11 years are anaemic if Hb level is below 11.5 g/dl and children from age 12 to 14 years are considered anaemic if Hb is below 12.0 g/dl^30^. The cause of anaemia is dependent on the part of the world a child lives.

Timely diagnosis and immediate treatment have been advised by WHO as major strategies in managing malaria and anaemia and in reduction of high mortality in most prevalent regions. Considering the strong correlation between malaria infection and anaemia, both microscopy and Rapid Diagnostic Tests were approved for the diagnosis of the two diseases in field surveys^31^. In this work, the upshot of interest was basically on the result of malaria rapid test and anaemic status as binary indicators of the presence of malaria and anaemia in a child’s blood sample respectively, where 1 denotes the presence of malaria or anaemia and 0 otherwise. The yearly distribution of malaria and anaemia during the last two weeks before the interview is as follows; in 2010, 5056 and 5147 were tested for anaemia and malaria, where for anaemia, 3512 children tested negative and 1544 tested positive while for malaria, 2719 tested negative and 2428 tested positive. While in 2015, 6021 and 6025 were tested for anaemia and malaria respectively, this has in the record that 4062 tested negative and 1959 tested positive for anaemia, on the other hand, 3399 tested negative and 2626 tested positive for malaria.

**Figure 1:**
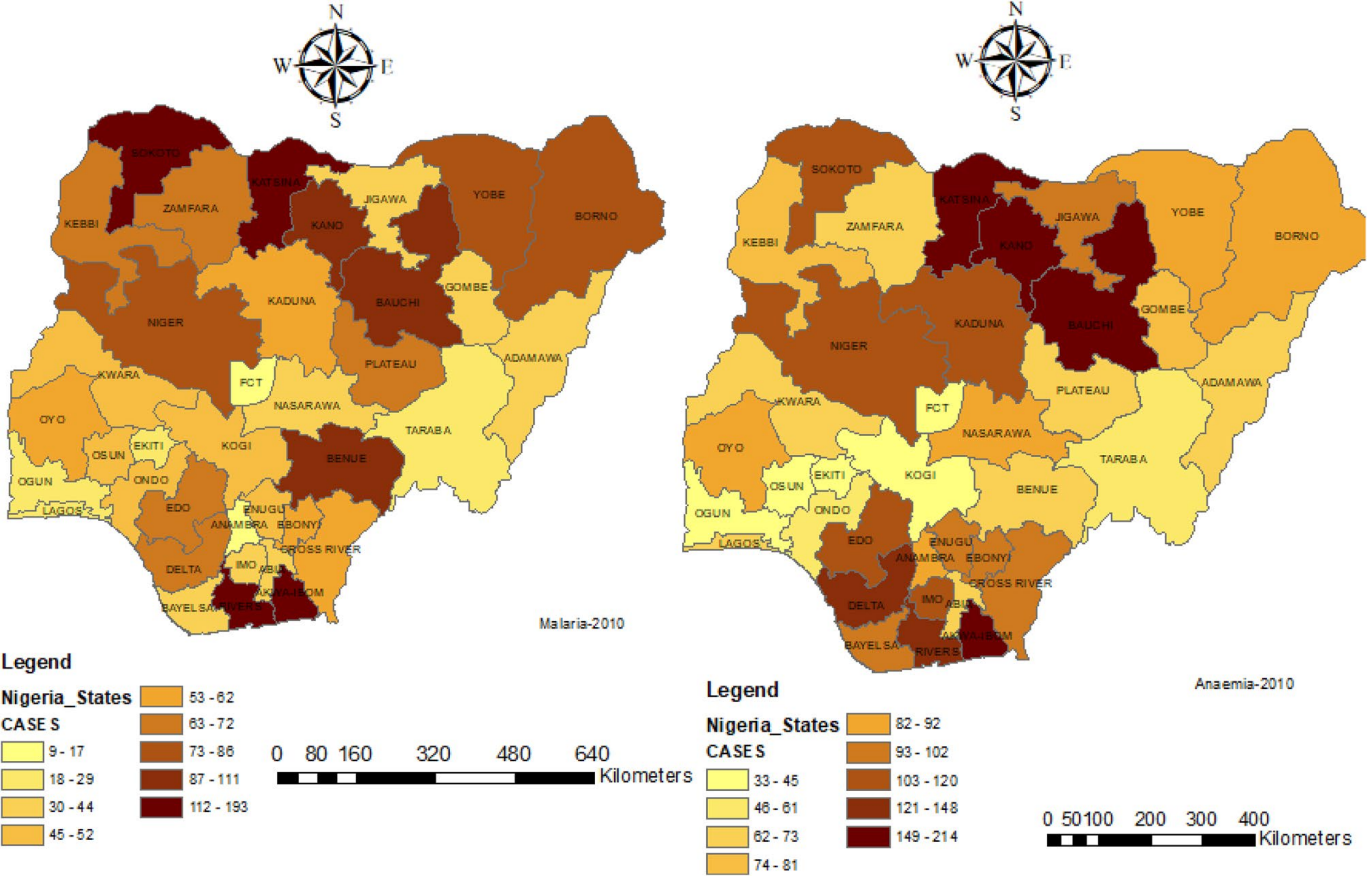
The 37 states including the capital territory of Nigeria showing the 2010 prevalence of malaria and anaemia of under-5 years of children.

**Figure 2:**
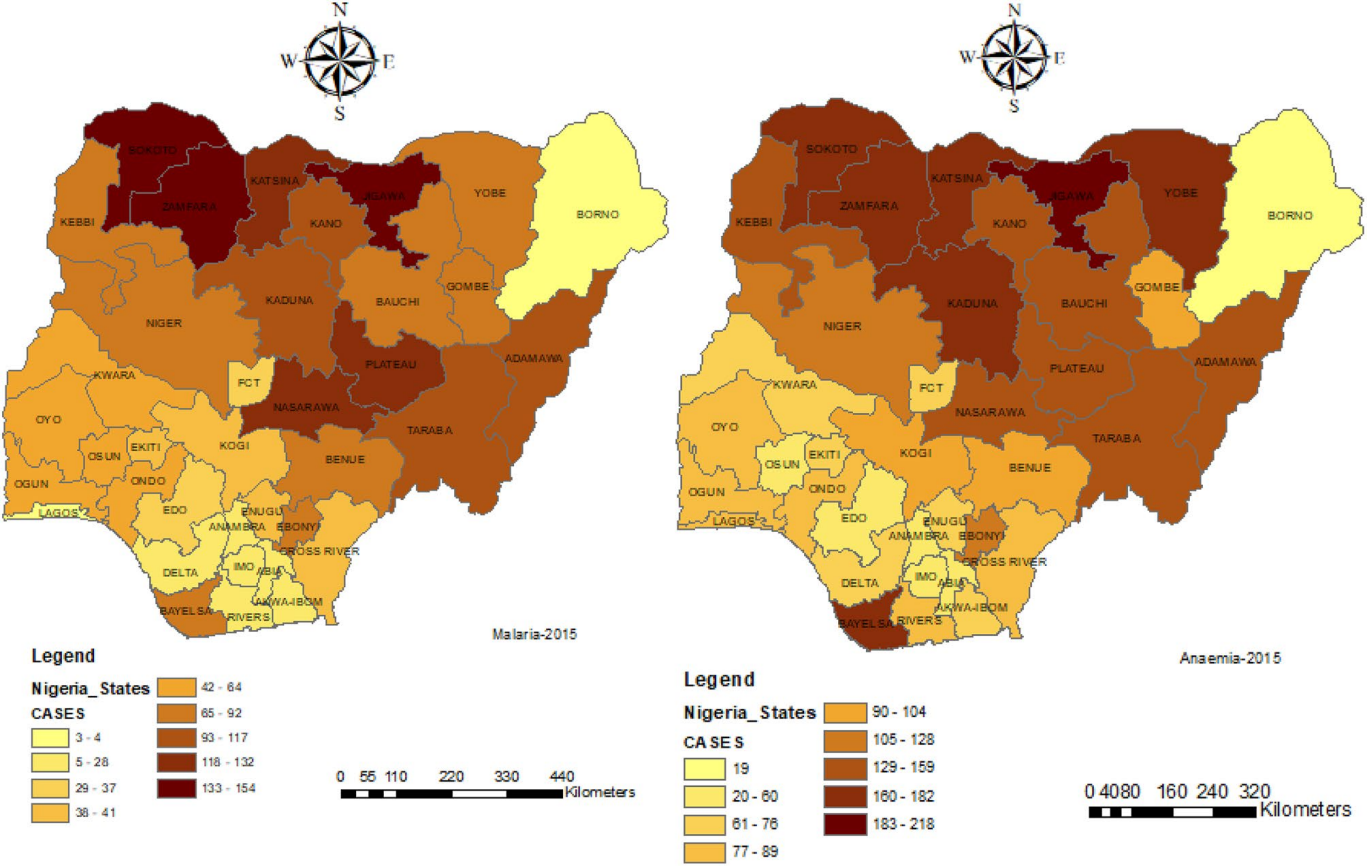
The 37 states including the capital territory of Nigeria showing the 2015 prevalence of malaria and anaemia of under-5 years of children.

Figures 1 and 2 present the map of Nigeria with its 37 states including the capital territory. Each of the maps displays the prevalence of malaria and anaemia in 2010 and 2015 respectively.

### Independent variables

In this study, the independent variables considered included some demographic, socio-economic and geographical variables which were based on the previous studies^2,11,32^. These variables comprise type of place of residence, source of drinking water, type of toilet facility, presence of electricity, own a radio, own a television, main floor material, main wall material, main roof material, wealth index, child’s age in months, sex, mother’s highest educational level while state and region were included as geographical variables. The selected variables in Table 1 were based on DHS and MIS data sets as well as relevant literature.

**Table 1:** Selected variable (risk factors) of malaria and anaemia among children aged 0 to 59 months.

Demographic factors	Socio-economic factors	Geographical factors
Type of place of residence	Wealth index	State
	Mother's highest educational level	
	Has radio	
	Has television	
	Source of drinking water	
	Type of toilet facility	
	Sex	
	Child's age in months	
	Main roof material	
	Main floor material	
	Main wall material	
	Has electricity	

### Methods

Here, we explained the Bayesian spatial models used to estimate the spread and the risk factors of malaria and anaemia in the 37 states including the Federal Capital in Nigeria.

#### Spatial model

Descriptive statistics approach was used to analyze the independent variables of the study sample in the form of descriptive table and simple percentages, while each of the dependent variables was described in the form of maps. For malaria or anaemia amongst children under age 5 years, their respective relationships with the independent variables were tested using the chi-square association analysis. Thereafter, a binary logistic regression was used to study the relationship between the independent variables and malaria or anaemia. A stepwise backward selection was done to pick the factors that have significant relationship with malaria or anaemia amongst children under 5 years. While for adjustment of clustering, common causes and sampling weights were carried out using the already weighting factors constructed by measure DHS.

For the spatial study of malaria and anemia data, let ${y}_{ik}$ be a binary malaria or anaemic status of child $i, i=\mathrm{1,2},\dots ,{n}_{k}$ where ${n}_{k}$ is the number of children in state $k$, $k=\mathrm{1,2},\dots ,37$ that have malaria or anaemia. Then the binary response follows as:

$$y_{ik} = \left\{ {\begin{array}{*{20}ll} {1,} & \quad {{\text{If child}}\;i\;{\text{in state}}\;k\;{\text{has malaria or is anaemic}}} \\ {0,} & \quad {{\text{Otherwise}}} \\ \end{array} } \right.$$

The ${y}_{ik}$ is assumed to follow a Bernoulli distribution with likelihood function defined as

$${y}_{ik}\sim Bernoulli \left({\theta }_{ik}\right), k=1,\dots ,37, i=1,\dots ,n$$

where ${\theta }_{ik}=P\left({y}_{ik}=1\right)$ are unknown probabilities and $E\left({y}_{ik}\right)={\theta }_{ik}$ is related to predictor through a link function

1 $$logit\left({\theta }_{ik}\right)=log\left(\frac{P\left({\theta }_{ik}=1\right)}{1-P\left({\theta }_{ik}=1\right)}\right)={\eta }_{ik}={x}_{ik}^{^{\prime}}\beta$$

the vector ${x}_{ik}={\left(1,{x}_{ik1},\dots ,{x}_{ikp}\right)}^{^{\prime}}$ are categorical and continuous variables and the vector of regression coefficients is $\beta =\left({\beta }_{0},{\beta }_{1},\dots ,{\beta }_{p}\right)$. This model accepts only a parametric form of categorical variables. To account for more flexible approach, the linear predictor ${\eta }_{ik}$ of Eq. (1) will be extended. Therefore, the model complexity is increased by including different forms of variables. The logistic regression model is then extended to give room for area-specific random effects by substituting the linear predictor ${\eta }_{ik}$ in Eq. (1) with a geoadditive predictor. These random effects are put in the model to take care of extra variation. Consequently, to incorporate unobserved influential factors that changes across the states, the structured random effects is accounted for by the model and it is defined as:

2 $${\eta }_{ik}={x}_{ik}^{^{\prime}}\beta +{f}_{str}\left({s}_{i}\right)$$

On a general note, the spatial effects of an areal unit can be modelled using the CAR model. Particularly in the second stage of hierarchical models, they are used to specify some classes of the model. in this work, the CAR model is expressed as follows. Let $u=\left({u}_{1},\dots ,{u}_{n}\right)$ be the vector of univariate random variables in relation to the observed spatial unit understudy and represent $\left\{\partial \left(i\right):i=1,\dots ,n\right\}$ as the states sharing the same border with state $i$. This means, for any $i, k=1,\dots ,n, k\in \partial \left(i\right)$ if only $i\in \partial \left(k\right)$ and $i\notin \partial \left(i\right)$ must be satisfied.

Therefore, suppose the conditional density of ${u}_{i}, i=1,\dots ,n,$ follows the conditional normal variable defined as;

3 $${u}_{i}/{u}_{k},\left(i\ne k\right)\sim N\left({\mu }_{i}+\sum_{k\in \delta \left(i\right)}{c}_{ik}\left({u}_{k}-{\mu }_{k}\right),{d}_{i}^{2}\right), i,k=1,\dots ,n$$

where ${u}_{k}$ is the mean for state $k, {\mu }_{i}$ is the spatial trend at location $i$ and ${d}_{i}^{2}={\rho }_{u}^{2}/\partial \left(i\right)$ is the conditional variance of the $i$ th state, which depends on the number of neighbours. Therefore, the size of the variance for the current state is determined by the number of state neighbours. ${c}_{ik}$ is the spatial dependence parameters for $i=1,\dots ,n,$ such that ${c}_{ii}=0$ for all $i$’s while ${\rho }_{u}^{2}$ denotes the variance parameter that controls the differences between spatial similarity? Particularly, the quantity ${c}_{ik}$ captures spatial dependency. The matrix form of Eq. (3) is given by Cressie^33^

4 $$u\sim N\left(\mu ,{B}^{-1}L\right),$$

which denotes the joint distribution of $u$, where $B=\left(I-C\right),$ with $C={\left[{c}_{ik}\right]}_{n\times n},$ $\mu =\left({\mu }_{1},\dots ,{\mu }_{n}\right)$ and $L=diag\left({d}_{1}^{2},\dots ,{d}_{n}^{2}\right)$ is an $n\times n$ diagonal matrix. Equation (4) is correct if $B$ can be inverted and ${B}^{-1}L$ is symmetric and the conditional constraints ${c}_{ik}{d}_{k}^{2}={c}_{ki}{d}_{i}^{2}$ for all $i\ne k$, and must be positive definite.

The elements of invertible matrix $B$ is expressed as;

5 $${b}_{\left(ik\right)}=\left\{\begin{array}{ll}1 & \quad for \; i=k\\ -{c}_{ik} & \quad for \; k\in \partial \left(i\right),\\ 0, & \quad otherwise,\end{array}\right.$$

To get a valid joint distribution, the covariance matrix in Eq. $\left(4\right)$ must be symmetric and positive definite as mentioned above. Then the symmetric weighted adjacency matrix will be $W=\left({W}_{ik}\right)$, and set ${c}_{ik}=\varnothing {W}_{ik}$ where

6 $$W_{ik} = \left\{ {\begin{array}{*{20}ll} 1 & {\text{if i and k share the same border}} \\ 0 & {{\text{otherwise}},} \\ \end{array} } \right.$$

and the properness of the distribution is controlled by the parameter $\varnothing$.

Also, to account for unobserved heterogeneity within each state, the unstructured random effects is considered, and the model is expressed as:

7 $${\eta }_{ik}={x}_{ik}^{^{\prime}}\beta +{f}_{unstr}\left({s}_{i}\right)$$

#### The Besag, York, and Mollie (BYM) model

Besag, York and Mollie (BYM) model proposed by^34^ is the most popularly used tool under the spatial Bayesian hierarchical models for disease mapping. The BYM comprises two random components, i.e., spatially structured $u$ and spatially unstructured $v$ components, which are included in the log-linear model for relative risk. By inclusion of these random effects, the smoothing of the relative risk at the state level is guaranteed. While spatially structured component $u$ is correlation of neighbouring spatial units, the spatially unstructured component $v$ is the uncorrelated extra variation. Also, note that the vectors $u$ and $v$ have individual unit random effects ${u}_{i}\left(i=1,\dots ,n\right)$ and ${v}_{i}\left(i=1,\dots ,n\right)$ respectively.

Therefore, Eq. (3) is extended to convolution model by adding both structured and unstructured random effects as follows:

8 $${\eta }_{ik}={x}_{ik}^{^{\prime}}\beta +{{f}_{str}\left({s}_{i}\right)+f}_{unstr}\left({s}_{i}\right)$$

In Eqs. (2)–(4), ${x}^{^{\prime}}$ is a k-dimensional row-vector of covariates with $\beta$ as the corresponding vector of regression coefficients. ${u}_{i}$ is the spatially structured random effect (correlated heterogeneity) and ${v}_{i}$ is the spatially unstructured effect (uncorrelated heterogeneity). On assumption^34^, stated that two random effects are independent and need a specification of independent priors. For the spatially unstructured ${v}_{i}$, the priors distribution model is assumed to follow a normal distribution with a vector of mean 0 and a variance–covariance matrix ${\sigma }^{2}I$, where $I$ is the identity matrix and ${\sigma }^{2}>0$ is unknown. Following the argument of^34^, the prior of the spatial component is assumed to be represented by a Markov Gaussian field or conditional Gaussian autoregressive model. Therefore, excluding the $kth$ state, let ${u}_{-k}$ denote the vector of effects, and then we assume that

9 $${u}_{k}|{u}_{-k}\sim N\left(\frac{1}{{n}_{k}}\sum_{e\sim k}{u}_{e},\frac{{\tau }_{u}^{2}}{{n}_{k}}\right)$$

where ${n}_{k}$ is the number of neighbourhoods of state $k$, while $e\sim k$ are all units $e$ neighbourhoods of state $k$ and ${\tau }_{u}$ is the standard deviation parameter. Finally, the inverse gamma hyperpriors is assumed for the variance of the normal priors.

The posterior distributions of the parameters were estimated using Integrated Nested Laplace Approximation (INLA)^35^ in R. This is because it is a better approach compared to Markov Chain Monte Carlo sampling and approximate Bayesian inference^36^. The Deviance Information Criteria (DIC) is calculated as; $DIC=\overline{D }\left(\theta \right)+Dp$, where $\overline{D }$ is the posterior mean of the deviance that measures the goodness of fit while $Dp$ is the effective number of parameters in the model. Based on the $DIC$, the final model was selected and the model with the smallest DIC was taken as a better fit^37^.

### Ethics approval and consent to participate

Secondary data was used for this study. The data is available for research purposes from the Demography and Health Survey website. Therefore, formal ethical approval is not applicable for this study. All methods were carried out in accordance with relevant guidelines and regulations.

## Results

Continuous variables with non-linear effect were studied; nonetheless, age in months was the only variable that showed a significant non-linear effect on the log-odds of a child's Malaria result and anaemic status. Therefore, this is the only non-linear effect incorporated in the fitted model, while the other independent variables were added as a linear fixed effect.

Table 2 presents the 2010 and 2015 percentages of children examined for malaria and anaemia. Individual data records of malaria and anaemia were constructed two weeks before the interview for 5147 and 5056 children between the age 0 to 5 years old in 2010, respectively. The same was done in 2015 with 6025 children for malaria and 6021 children for anaemia. In child’s age in months, group 6 (age 51–59 months) had the highest record of the two diseases in both years and it was more in male children (2010: anaemia—50.5%, malaria—50.6%. 2015: anaemia—50.4%, malaria—50.4%). Most of these children lived in rural areas where there is no electricity, no television, no good toilet facilities, and the source of drinking water is not healthy. Regarding region, in both years, Northwest had the highest record of the two diseases with majority of illiterate mothers (2010: anaemia—45.6%, malaria—46.1%. 2015: anaemia—43.5% and malaria—43.5%) and low standard of living, also poor building materials.

**Table 2:** Percentage of malaria and anaemia with the p-value of under age 5 years children in 2010 and 2015 for each variable.

Individual characteristics	2010				2015			
	Malaria (%)	p-value	Anaemia (%)	p-value	Malaria (%)	p-value	Anaemia (%)	p-value
Factors								
**Child’s age in months**		< 0.001		< 0.001		< 0.001		< 0.001
1	18.1		18.1		16.2		16.2	
2	13.7		13.8		15.5		15.5	
3	17.6		17.5		16.5		16.5	
4	17.2		17.1		18.1		18.1	
5	16.5		16.7		16.2		16.2	
6	16.9		16.9		17.6		17.6	
**Sex**		0.001		0.001		0.063		0.001
Male	50.6		50.5		50.4		50.4	
Female	49.4		49.5		49.6		49.6	
**Region**		0.004		< 0.001		< 0.001		0.145
North Central	17.3		17.5		20		20	
North East	17.7		17.9		19.2		19.2	
North West	24.5		23.3		25		25	
South East	12.4		12.6		9.9		9.8	
South South	18.7		19.1		12.8		12.9	
South West	9.4		9.6		13.1		13.1	
**Electricity**		< 0.001		< 0.001		< 0.001		< 0.001
No	52.8		52.9		52.1		52.1	
Yes	47.2		47.1		47.9		47.9	
**Wealth index**		< 0.001		< 0.001		< 0.001		< 0.001
Poor	36.9		36.7		40.2		40.2	
Middle	22.6		22.5		20.9		20.9	
Rich	40.5		40.8		38.9		38.9	
**Mother’s educational level**		< 0.001		< 0.001		< 0.001		< 0.001
No education	46.1		45.6		43.5		43.5	
Primary	20.7		20.8		18.3		18.3	
Secondary	28.4		28.7		30.4		30.4	
Higher	4.8		4.9		7.7		7.7	
**Has radio**		< 0.001		< 0.001		< 0.001		< 0.001
No	28.7		28.6		39.8		39.8	
Yes	71.3		71.4		60.2		60.2	
**Type of place of residence**		< 0.001		< 0.001		< 0.001		< 0.001
Urban	27.3		27.3		33.9		33.9	
Rural	72.7		72.7		66.1		66.1	
**Has television (ref = no)**	59		58.9		54.2		54.2	
Yes	41		41.1		45.8		45.8	
**Source of drinking water**		< 0.001		0.324		0.064		< 0.001
Tap/other water	38.5		38.6		39.9		40.9	
Well water	61.5		61.4		61.1		59.1	
**Type of toilet facility**		< 0.001		< 0.001		< 0.001		< 0.001
Flush/other toilet	45.6		46.3		50		50	
Pit toilet	54.4		53.7		50		50	
**Main roof material**		< 0.001		0.034		< 0.001		< 0.001
Wood/other	34.9		34.9		28.8		28.8	
Zinc/metal	65.1		65.1		71.2		71.2	
**Main floor material**		< 0.001		0.452		< 0.001		< 0.001
Earth/other	49.2		48.9		44.2		44.2	
Cement/ceramics	50.8		51.1		55.8		55.8	
**Main wall material**		< 0.001		0.002		< 0.001		< 0.001
Wood/other	56.2		55.9		39.5		39.5	
Cement/bricks	43.8		44.1		60.5		60.5	

Table 3 contains the results of the DIC and the effective number of parameters *Dp* of 2010 and 2015 for each of the fitted models of malaria and anaemia. For 2010, the results of malaria are based on model 4 because it gave the least DIC, and this model contains both the structured and unstructured spatial effects. On the other hand, the results of anaemia are based on model 2 because it gave the least DIC. In 2015, the results of malaria are based on model 3 because it gave the least DIC. While the results of anaemia are based on model 4 because it gave the least DIC, and this model contains both the structured and unstructured spatial effect. It should be noted that in both 2010 and 2015, there was not much difference in the estimates of the fixed effects in the four models; nevertheless, the variables differed significantly.

**Table 3:** Model comparisons for malaria and anaemia.

	2010				2015			
	Model 1	Model 2	Model 3	Model 4	Model 1	Model 2	Model 3	Model 4
DIC (malaria)	5358.5	5014.26	5014.26	5014.01	5774.77	5773.55	5770.02	5770.23
DIC (anaemia)	4725.58	4542.03	4543.82	4542.15	5801.67	5793.63	5794.57	5793.43
Dp (malaria)	26.91	54.26	53.2	54.01	57.46	52.68	51	51.43
Dp (anaemia)	26.89	53.68	53.78	53.72	57.52	49.35	47.8	49.13

Table 4 presents the adjusted posterior odds ratio estimates (AOR) and 95% credible interval for the random effects included in the Bayesian hierarchical logistic regression model. In 2010, there was a significant increase in the odds of malaria for children aged 2 (15 to 23 months), 3 (24 to 32 months), 4 (33 to 41 months), 5 (42 to 50 months) and 6 (51 to 59 months) relative to children in aged 1 (6 to 14 months). There is a significant increase in the odds of malaria among children who have anaemia or reside in rural areas^2^. On the other hand, the household that has electricity had significantly lower odds of malaria compared to a household with no electricity. In the same vein, the odds of malaria decrease significantly among those who use well water relative to those who use tap/other and those who use pit toilet relative to those who use flush/other toilet. Furthermore, lower odds of malaria were suggested for mothers with higher educational level (Secondary and Higher education) and wealth index (Rich). Female children had an increased odds of malaria than male children; however, these odds were not significant; the same applies to main wall material and households that had a radio. With respect to main roofing material, main floor material and households that had a television, there was no significant decrease in odds of malaria. While in 2015, the odds of malaria for children aged 2 (15 to 23 months), 3 (24 to 32 months), 4 (33 to 41 months), 5 (42 to 50 months) and 6 (51 to 59 months) shows a significant increase relative to children aged 1 (6 to 14 months). Similarly, there is a significant increase in the odds of malaria among children who have anaemia or live in rural areas with zinc/metal roof. On the other hand, the odds of malaria decrease significantly among those who use pit toilet relative to those who use flush/other toilet. Furthermore, the results suggested that children from mothers with higher educational level (Secondary and Higher education) and wealth index (Middle and Rich) have lower odds of malaria. Female children had higher odds of malaria than male children; however, these odds were not significant; the same applies to electricity, main floor material, main wall material and household that has a radio. There was a non-significant increase in odds of malaria with respect to source of drinking water and household that has television.

**Table 4:** 95% credible interval and adjusted posterior odd ratios estimates (AOR) of malaria.

Variable	2010		2015	
	AOR	95% CI	AOR	95% CI
Intercept	0.468	(0.318, 0.685)	0.185	(0.133, 0.258)
**Child’s age in months (grouped) (ref = 1)**				
2	1.315*	(1.035, 1.670)	1.616*	(1.293, 2.020)
3	1.818*	(1.453, 2.275)	2.194*	(1.760, 2.738)
4	2.139*	(1.704, 2.688)	2.733*	(2.197, 3.405)
5	2.304*	(1.830, 2.905)	3.447*	(2.746, 4.334)
6	2.444*	(1.938, 3.086)	4.149*	(3.310, 5.211)
**Has electricity (ref = no)**				
Yes	0.773*	(0.631, 0.949)	0.773	(0.631, 1.064)
**Source of drinking water (ref = tap/other water)**				
Well water	0.810*	(0.696, 0.942)	1.122	(0.971, 1.295)
**Type of toilet facility (ref = flush/other toilet)**				
Pit toilet	0.823*	(0.697, 0.973)	0.718*	(0.619, 0.833)
**Anaemic status (ref = no)**				
Yes	2.583*	(2.198, 3.039)	3.345*	(2.880, 3.890)
**Wealth index (ref = poor)**				
Middle	0.887	(0.677, 1.163)	0.710*	(0.568, 0.887)
Rich	0.634*	(0.415, 0.967)	0.383*	(0.277, 0.529)
**Sex (ref = male)**				
Female	1.093	(0.954, 1.252)	0.956	(0.842, 1.085)
**Mother’s educational level (ref = no education)**				
Primary	1.062	(0.851, 1.324)	0.788*	(0.652, 0.953)
Secondary	0.684*	(0.538, 0.869)	0.790*	(0.646, 0.967)
Higher	0.529*	(0.355, 0.783)	0.389*	(0.268, 0.558)
**Main roof material (ref = wood/other)**				
Zinc/metal	0.88	(0.734, 1.055)	1.214*	(1.016, 1.450)
**Main floor material (ref = earth/other)**				
Concrete/ceramics	0.86	(0.696, 1.063)	0.911	(0.769, 1.080)
**Main wall material (ref = wood/other)**				
Cement/bricks	1.034	(0.808, 1.323)	0.968	(0.800, 1.171)
**Has radio (ref = no)**				
Yes	1.116	(0.948, 1.315)	0.902	(0.783, 1.039)
**Type of place of residence (ref = urban)**				
Rural	1.348*	(1.117, 1.627)	1.889*	(1.568, 2.277)
**Has television (ref = no)**				
Yes	0.872	(0.698, 1.090)	1	(0.807, 1.240)

*means significant

**Figure 3:**
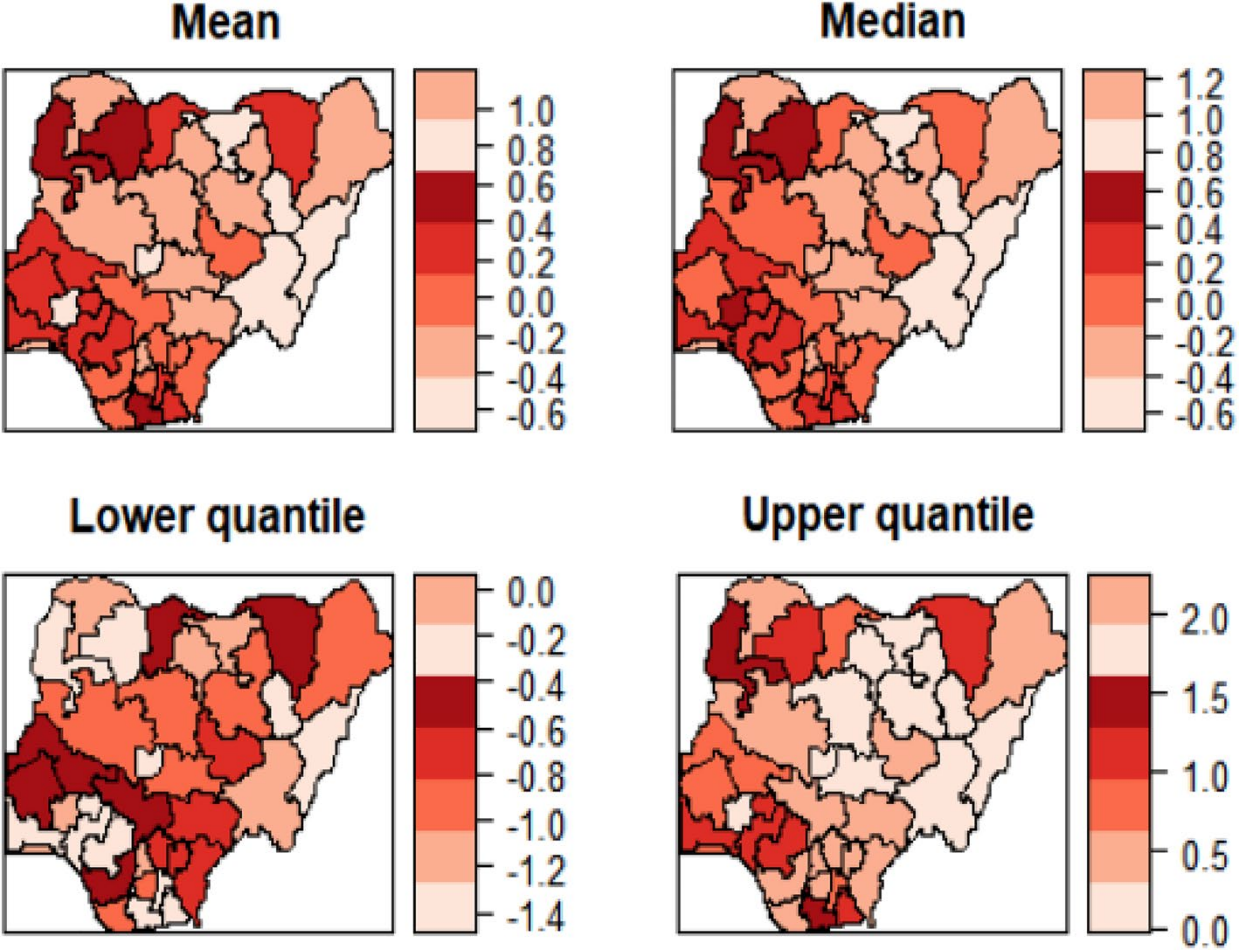
Nigeria state-level estimated posterior mean of the structured spatial effect maps: (a) Mean, (b) Median, (c) 25% quantile and 97.5% quantile for 2010.

**Figure 4:**
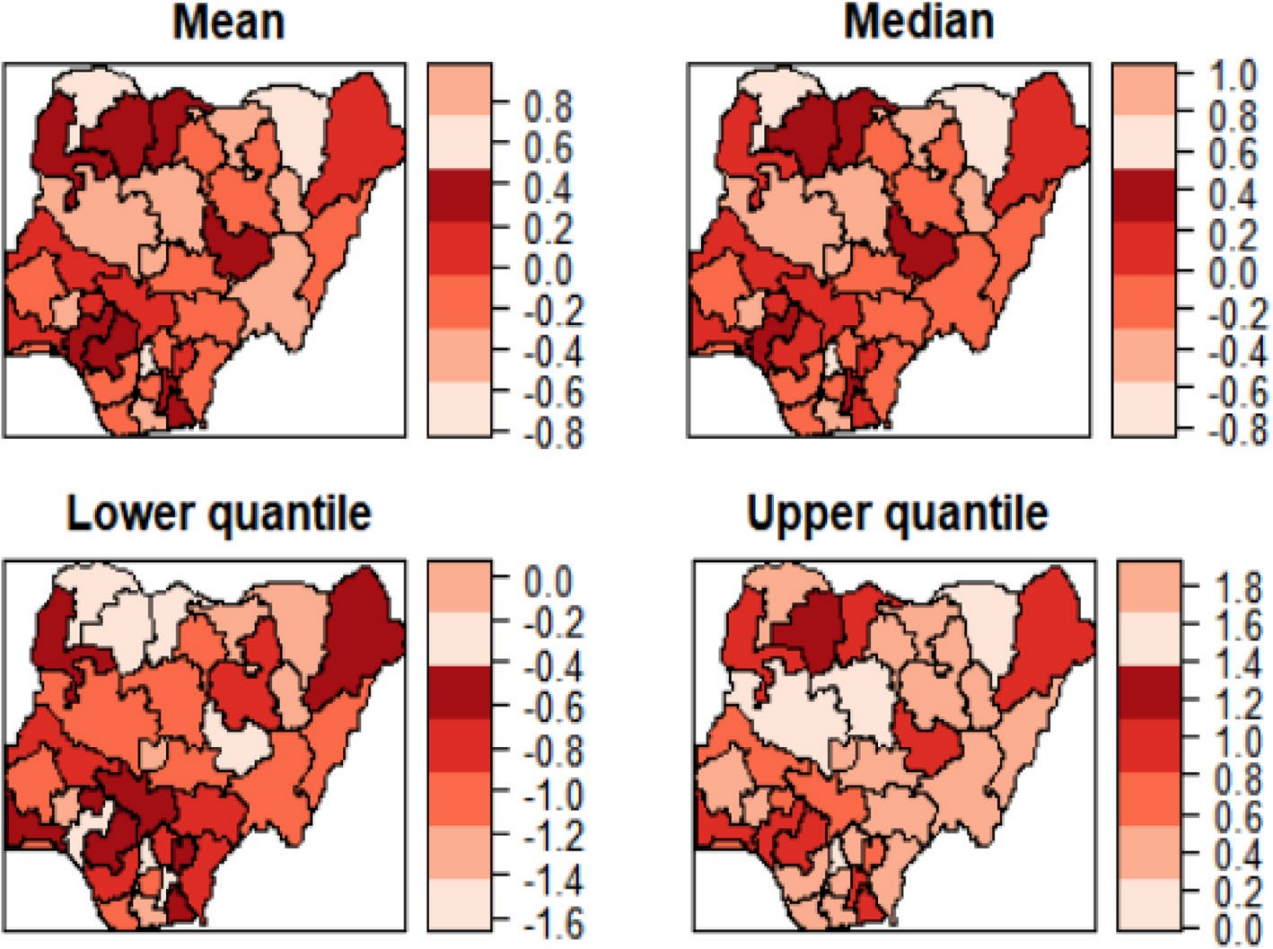
Nigeria state-level **e**stimated posterior mean of the unstructured spatial effect maps: (a) Mean, (b) Median, (c) 25% quantile and 97.5% quantile for 2010.

Figures 3 and 4 show the estimated mean, median, 25% quantile and 95% quantile of the structured and unstructured spatial effects on the log-odds of malaria, respectively. Lower odds of malaria are associated with the eastern regions because they have a negative spatial effect, while higher odds of malaria are associated with the northern regions because they have a positive spatial effect. This could be attributed to several factors among them being that northern women are less educated and therefore are not well informed about this disease. Also, most northern women are rural dwellers and so lack the knowledge of malaria protection role of modern housing. In comparison, the structured spatial effect that ranged from − 0.6 to 1.0 for mean, − 0.6 to 1.2 for median, − 1.4 to 0.0 for 25% quantile and 0.0 to 2.0 for 95% quantile is seen to be stronger than unstructured spatial effect that ranged from − 0.8 to 0.8 for mean, − 0.8 to 1.0 for median, − 1.6 to 0.0 for 25% quantile and 0.0 to 1.8 for 95% quantile. From this, the structured spatially correlated effect was stronger than the unstructured spatial effect, indicating that there is a similarity in the effect a particular region has on the risk of malaria and her neighbouring regions. This shows that there is the possibility of demographic, socio-economic, and geographical factors that go beyond the boundaries of the regions playing a significant role in childhood malaria. Also, the strong relationship between malaria and anaemia in Nigeria could be the result of the homogenous outcome of spatial effect on childhood malaria in the country, and this was explained by the inclusion of child’s anaemic status.

The adjusted posterior odds ratio estimates (AOR) and 95% credible interval for the random effects incorporated in the Bayesian hierarchical logistic regression model are presented in Table 5. In 2010, the odds of anaemia increased significantly for children aged 3 (24 to 32 months), 4 (33 to 41 months), 5 (42 to 50 months) and 6 (51 to 59 months) relative to children aged 1 (6 to 14 months). The odds of anaemia increase significantly among children who have malaria or live in rural areas. In the same vein, regarding wealth index, children from the middle and rich household had a significant increase in the odds of anaemia relative to children from a poor households. On the other hand, the household that has electricity had non significantly lesser odds of anaemia compared to a household with no electricity. There was a non-significant decrease in the odds of anaemia among those who use well water relative to those who use tap/other and those who use pit toilet relative to those who use flush/other toilet. Furthermore, mothers with higher educational level (Secondary and Higher education) had lower odds of anaemia but not significant. The odds of anaemia increased significantly for female children than male children. The same applies to households that had a radio and television. No significant decrease in the odds of anaemia was recorded with respect to main roof material, main floor material and main wall material. In 2015, the odds of anaemia decreased significantly for children aged 3 (24 to 32 months), 4 (33 to 41 months), 5 (42 to 50 months) and 6 (51 to 59 months) relative to children aged 1 (6 to 14 months). Also, the odds of anaemia among children who have malaria as well live in rural areas and use well water and pit toilet increase significantly. On the other hand, regarding wealth index, electricity and concrete/ceramics floor, non-significant increase in the odds of anaemia was noted. The odds of anaemia among female children relative to male children and those who have television to those who do not have decreased non-significantly. With respect to main roof material, main floor material and main wall material and the household that have radio, the odds of anaemia did not decrease significantly.

**Table 5:** 95% credible interval and adjusted posterior odd ratios estimates (AOR) of anaemia.

Variable	2010		2015	
	AOR	95% CI	AOR	95% CI
Intercept	4.184	(2.755, 6.396)	3.23	(2.338, 4.468)
**Child’s age in months (grouped) (ref = 1)**				
2	0.892	(0.682, 1.684)	0.914	(0.722, 1.158)
3	0.693*	(0.539, 0.890)	0.529*	(0.421, 0.664)
4	0.429*	(0.335, 0.549)	0.378*	(0.303, 0.470)
5	0.361*	(0.281, 0.463)	0.303*	(0.241, 0.380)
6	0.340*	(0.265, 0.435)	0.271*	(0.216, 0.340)
**Has electricity (ref = no)**				
Yes	0.98	(0.783, 1.227)	1.003	(0.825, 1.219)
**Source of drinking water (ref = tap/other water)**				
Well water	0.98	(0.832, 1.153)	1.205*	(1.049, 1.386)
**Type of toilet facility (ref = flush/other toilet)**				
Pit toilet	0.961	(0.806, 1.145)	1.191*	(1.031, 1.377)
**Result of malaria rapid test (ref = no)**				
Yes	2.606*	(2.216, 3.068)	3.386*	(2.916, 3.936)
**Wealth index (ref = poor)**				
Middle	1.465*	(1.093, 1.964)	1.069	(0.840, 1.361)
Rich	1.752*	(1.106, 2.778)	1.013	(0.728, 1.408)
**Sex (ref = male)**				
Female	0.761*	(0.658, 0.877)	0.793*	(0.699, 0.901)
**Mother’s educational level (ref = no education)**				
Primary	0.814	(0.642, 1.033)	0.877	(0.720, 1.068)
Secondary	0.87	(0.676, 1.119)	0.746*	(0.612, 0.910)
Higher	0.937	(0.633, 1.391)	0.578*	(0.436, 0.765)
**Main roof material (ref = wood/other)**				
Zinc/metal	0.904	(0.744, 1.099)	0.922	(0.769, 1.106)
**Main floor material (ref = earth/other)**				
Concrete/ceramics	1.003	(0.802, 1.253)	1.105	(0.860, 1.198)
**Main wall material (ref = wood/other)**				
Cement/bricks	0.881	(0.672, 1.152)	0.84	(0.685, 1.032)
**Has radio (ref = no)**				
Yes	0.659*	(0.548, 0.790)	0.877	(0.758, 1.014)
**Type of place of residence (ref = urban)**				
Rural	1.455*	(1.201, 1.763)	1.440*	(1.205, 1.719)
**Has television (ref = no)**				
Yes	0.660*	(0.516, 0.836)	0.908*	(0.731, 1.128)

*means significant

**Figure 5:**
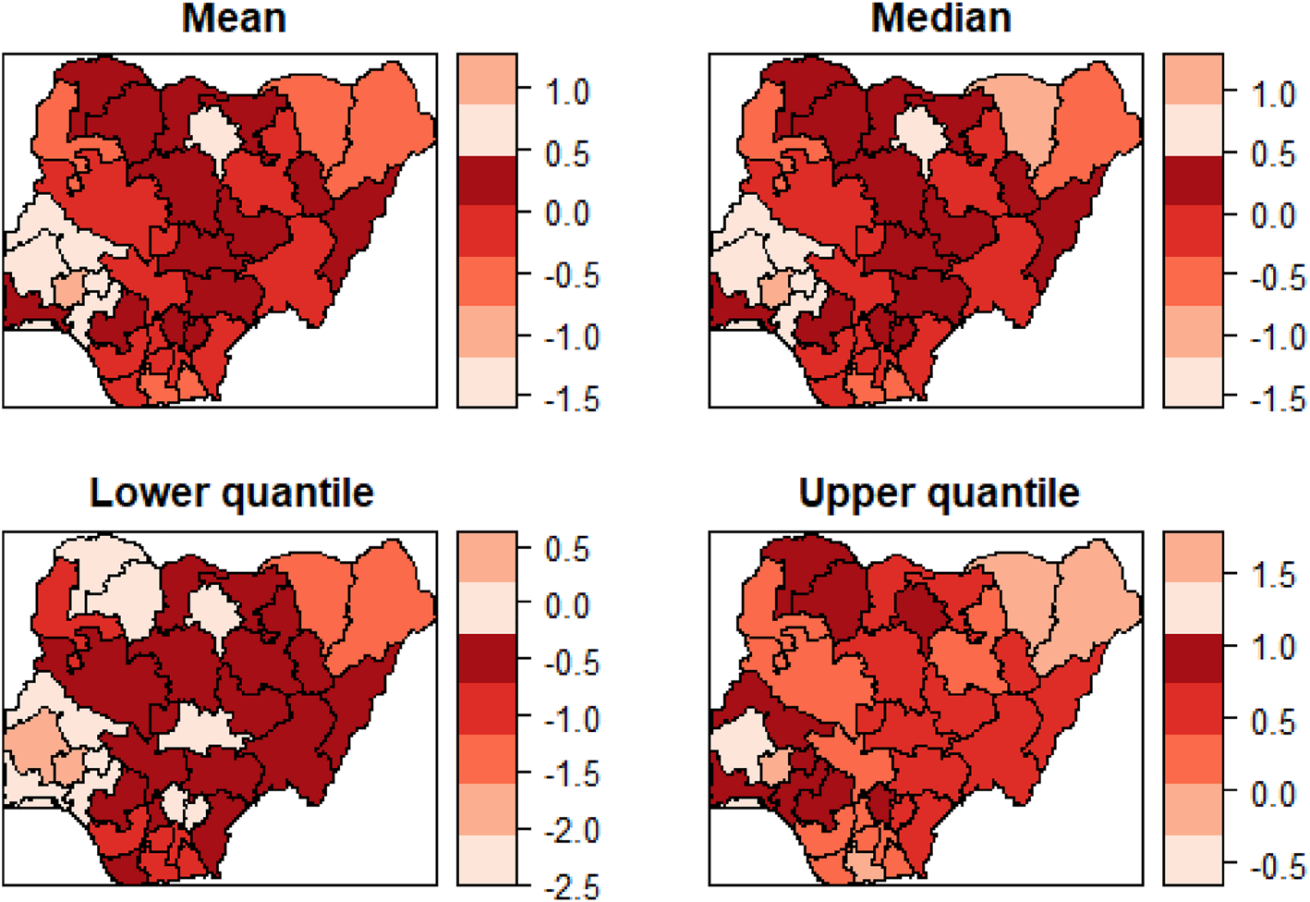
Nigeria state-level **e**stimated posterior mean of the structured spatial effect maps: (a) Mean, (b) Median, (c) 25% quantile and 97.5% quantile for 2015.

**Figure 6:**
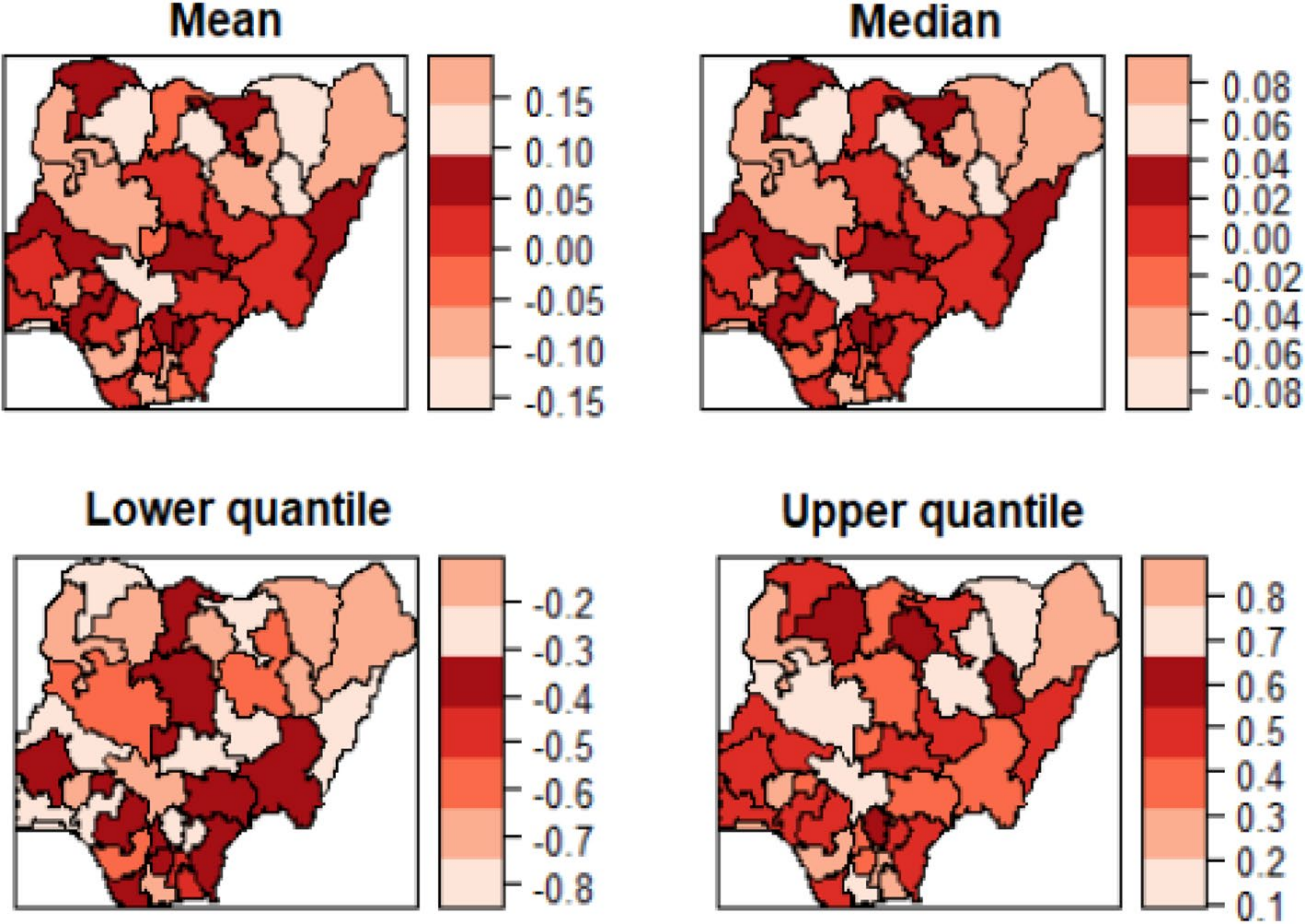
Nigeria state-level **e**stimated posterior mean of the unstructured spatial effect maps: (a) Mean, (b) Median, (c) 25% quantile and 97.5% quantile for 2015.

Figures 5 and 6 show the estimated mean, median, 25% quantile and 95% quantile of the structured and unstructured spatial effects on the log-odds of anaemia, respectively. Lower odds of anaemia were attributed to the northeast regions because they have a negative spatial effect, while a higher odd of anaemia was ascribed to the core north regions because they have a positive spatial effect. This is because most northern women are less educated and therefore are not well informed about this disease. Also, most northern women are rural dwellers and so lack the knowledge of anaemia protection role of modern housing. In comparison, the structured spatial effect that ranged from − 1.5 to 1.0 for mean, − 1.5 to 1.0 for median, − 2.5 to 0.5 for 25% quantile and − 0.5 to 1.5 for 95% quantile is estimated to be stronger than unstructured spatial effect that ranged from − 0.15 to 0.15 for mean, − 0.08 to 0.08 for median, − 0.8 to − 0.2 for 25% quantile and 0.1 to 0.8 for 95% quantile. Like in the previous maps, there is the similarity of factors that influences the risk of malaria which transcend boundaries among the neighbouring regions. In addition, the strong correlation between malaria and anaemia maybe because of the homogenous results of spatial effect on childhood anaemia. It was explained by adding a child’s malaria status.

## Discussion

In this study, a hierarchical Bayesian logistics regression model was adopted to investigate the spatial variation, socio-economic, demographic, and geographical factors of malaria and anaemia in children under age 5 years in Nigeria using two datasets. The spatial effect maps were generated from the available data, to identify the most endemic region. The result from this study is in line with the previous works^11,22,27,38^, that the risk of girls having malaria or anaemia is less, and an increase in wealth index and mother’s educational level decreases a child’s risk. It can be inferred that the more educated an individual is, the more aware and more understanding of health-related issues. Also, from the results, inefficient public health resource allocations and socio-economic conditions are strongly associated with geographical inequalities in health^27^. In the same vein, when individuals have a high income, access to good health care and nutritional food sources reduces the risk of the diseases. In both years, the type of place of residence is significantly associated with the two diseases. Children in rural areas tend to have higher rates of the diseases than their contemporaries^22,27,39^. In addition, female children had lower risk of having anaemia in both years while for malaria, female children had non-significant higher odds in 2010 and lower odds in 2015 compared to male children. This could be attributed to the customary practice that male children are most preferred by their fathers in Nigeria especially, in Igbo tribe than female children^40^. This could be the reason female children have the advantage of being breastfed for a longer period and also get better healthcare and nutrition, thereby improving their health status because they are left to be taken care of by their mothers^15^. There is a high possibility of denying the male children their mother’s care which may result in blood loss from injury, etcetera and higher levels of parasitosis which agree with previous studies^22,27,41^. As seen in Fig. 6, there is a significant association between malaria and anaemia in the two years. This suggests that a high rate of anaemia in this country is caused by malaria^11,17^. Children with a better source of drinking water are less affected by malaria and anaemia. It could be said that a better source of drinking water ensures good health for children which increases their body immunity. In accordance, the type of toilet facilities has a significant association with malaria which in turn causes anaemia. This is because when there is poor sanitation, It is possible for the facility to give a conducive environment for the breeding of mosquitoes which increases the risk of malaria parasites and then result in anaemia^11^. In this study, wall, floor, and roof materials were found not to be significantly associated with malaria or anaemia. But these factors have a high correlation with malaria, for this reason, many of these factors on childhood anaemia will be accounted for if the child’s malaria status is included^2,11,25^. It can be seen from our results that the vulnerability of under-5 malaria infection increases with age while anaemia infection decreases with age. For malaria, the reason could be that children within 6 to 23 months are protected through maternal immunity^8,27^. On the other hand, for anaemia, a disease controlling immunity develops as the child grows, as such, nearly all malaria infections are asymptotic by adolescents and adults, thus, there will be a decline in the prevalence of anaemia^17^.

The reason for considering more than one disease in two different years is to ascertain if both diseases are affected by the same demographical, socio-economic, or geographical factors. From this study, the effect of unstructured spatially correlation is seen to be moderately weak in contrast to structured spatial effect, signifying that the prevalence of malaria and anaemia in each of the years is the same among neighbouring states. In 2010, malaria and anaemia diseases were denser in all Nigerian states than in 2015. Also, in 2015, there is an obvious increase in mother’s educational levels which is a major reason for this change.

Notwithstanding the differences in the two years considered, there were similarities as seen in Figs. 1 and 2, both malaria and anaemia were predominant in North-West, North-Central and some part of South-East in the considered two datasets.

**Figure 7:**
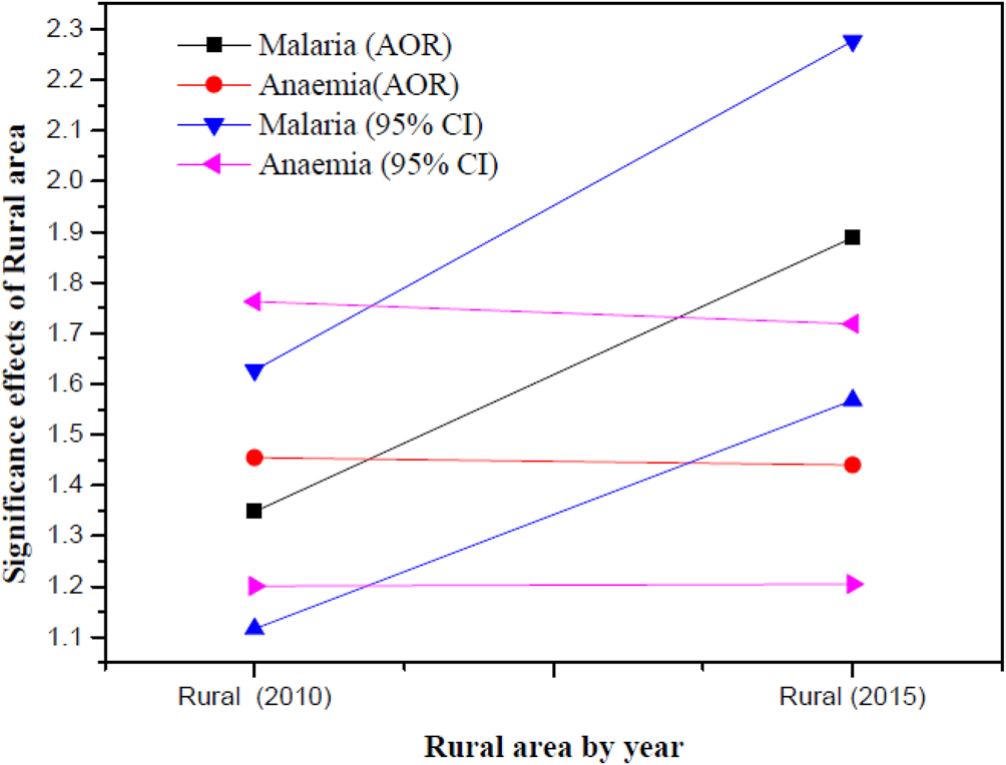
Estimated significance of non-linear effect of rural area, the posterior mean and the 95% credible intervals.

Figure 7 shows the significance of non-linear effect of rural area on malaria and anaemia. It can be deduced from our results that there was a significant increase in malaria in rural areas in both years while for anaemia, the effect increased but dropped slightly at some point. This is because as malaria infection increases, it gets to a point where individuals develop a disease controlling immunity such that all those who have malaria are asymptomatic and this diminishes the prevalence of anaemia^17^.

## Conclusion

The results from this study give clarity to the risk factors of malaria and anaemia in children between the age of 6 to 59 months and better understanding of strategies to mitigate the impacts of the diseases from a public health standpoint. For both diseases, children living in rural areas with illiterate mothers are the most affected. Malaria and anaemia control measures should study the spatial difference that is apparent in these regions. The odds of malaria and anaemia among children between the age of 6 to 59 months increase with child’s age, mother’s level of education, place of residence, source of drinking water, low income, type of toilet facility and presence of either of the diseases. Factors that contribute to spatial heterogeneity should be considered to focus on assessing local region-specific causes of anaemia and malaria in children.

## Data Availability

The data that support the findings of this study are available from Jecinta U. Ibeji, but restrictions apply to the availability of these data, which were used under license for the current study, and so are not publicly available. Data are however available from the authors upon reasonable request and with permission of Jecinta U. Ibeji.
